# Supplementary material for: Serum Progranulin Level Might Differentiate Non-IPF ILD from IPF
Source: Int J Mol Sci. 2023 May 24;24(11):9178. doi: 10.3390/ijms24119178 (PMC10252558; doi:10.3390/ijms24119178)
Supplement: Supplementary file 1 [file ijms-24-09178-s001.zip › ijms-2379925-supplementary.pdf]

Supplementary Material

Table S1. Stepwise multiple linear regression analysis of the potential predictors of serum PGRN level

**Linear regression analysis***					
Model		Beta	95.0% Confidence Interval for Beta		p value
			Lower Bound	Upper Bound	
Step 1	Age	0.550	0.323	0.552	**<0.001**
	Sex	0.427	8.375	16.936	**<0.001**
Step 2	Age	0.601	0.341	0.615	**<0.001**
	Sex	0.341	5.266	14.978	**<0.001**
	Antifibrotic treatment	0.009	-5.772	7.315	0.815
	Immunosupressive treatment	0.083	2.741	20.882	**0.011**
Step 3	Age	0.416	0.168	0.494	**<0.001**
	Sex	0.345	5.550	14.895	**<0.001**
	Antifibrotic treatment	-0.021	-8.373	4.679	0.576
	Immunosupressive treatment	0.045	-3.093	15.778	0.185
	ILD present	0.228	4.279	20.607	**0.003**
Step 4	Age	0.467	0.212	0.531	**<0.001**
	Sex	0.311	4.658	13.771	**<0.001**
	Antifibrotic treatment	0.081	-1.568	15.997	0.106
	Immunosupressive treatment	0.032	-4.653	13.706	0.330
	ILD present	0.262	6.321	22.261	**0.001**
	IPF	-0.162	-21.928	-4.234	**0.004**
Step 5	Age	0.353	0.125	0.436	**<0.001**
	Sex	0.195	1.207	10.350	**0.014**
	Antifibrotic treatment	0.078	-1.259	15.107	0.096
	Immunosupressive treatment	0.015	-6.572	10.703	0.636
	ILD present	0.553	19.328	40.894	**<0.001**
	IPF	-0.053	-13.602	5.034	0.364
	UIP	-0.220	-19.966	-6.756	**<0.001**

*Dependent variable: PGRN level
